# Supplementary material for: InvSim algorithm for pre-computing airplane flight controls in limited-range autonomous missions, and demonstration via double-roll maneuver of Mirage III fighters
Source: Sci Rep. 2025 Jul 2;15:23382. doi: 10.1038/s41598-025-07639-6 (PMC12222780; doi:10.1038/s41598-025-07639-6)
Supplement: Supplementary file 1 — Supplementary Information. [file 41598_2025_7639_MOESM1_ESM.pdf]

$$\{\{\beta''(t) \to$$

$$\frac{1}{m\,V(t)}\bigl(0.5\,S\,\rho\,\bigl(1.\sin(\alpha(t))\sin(\beta(t))\bigl(\sin(\alpha(t))\,(C_{\mathrm{L0}} + C_{\mathrm{L}\alpha}\,\alpha(t)) + \cos(\alpha(t))\cos(\beta(t))\bigl(-K_{\mathrm{CD}}\,(C_{\mathrm{L0}} + C_{\mathrm{L}\alpha}\,\alpha(t))^2 - C_{\mathrm{D0}}\bigr) -$$

$$\cos(\alpha(t))\sin(\beta(t))\,C_{\mathrm{Y}\beta}\,\beta(t)\bigr)(t)\,\alpha'(t) - 1.\cos(\alpha(t))\sin(\beta(t))\bigl(-\cos(\alpha(t))\,(C_{\mathrm{L0}} + C_{\mathrm{L}\alpha}\,\alpha(t)) +$$

$$\cos(\beta(t))\sin(\alpha(t))\bigl(-K_{\mathrm{CD}}\,(C_{\mathrm{L0}} + C_{\mathrm{L}\alpha}\,\alpha(t))^2 - C_{\mathrm{D0}}\bigr) - \sin(\alpha(t))\sin(\beta(t))\,C_{\mathrm{Y}\beta}\,\beta(t)\bigr)(t)\,\alpha'(t) -$$

$$\sin(\beta(t))\bigl(\sin(\beta(t))\bigl(-K_{\mathrm{CD}}\,(C_{\mathrm{L0}} + C_{\mathrm{L}\alpha}\,\alpha(t))^2 - C_{\mathrm{D0}}\bigr) + \cos(\beta(t))\,C_{\mathrm{Y}\beta}\,\beta(t)\bigr)(t)\,\beta'(t) -$$

$$1.\cos(\alpha(t))\cos(\beta(t))\bigl(\sin(\alpha(t))\,(C_{\mathrm{L0}} + C_{\mathrm{L}\alpha}\,\alpha(t)) + \cos(\alpha(t))\cos(\beta(t))\bigl(-K_{\mathrm{CD}}\,(C_{\mathrm{L0}} + C_{\mathrm{L}\alpha}\,\alpha(t))^2 - C_{\mathrm{D0}}\bigr) -$$

$$\cos(\alpha(t))\sin(\beta(t))\,C_{\mathrm{Y}\beta}\,\beta(t)\bigr)(t)\,\beta'(t) - 1.\cos(\beta(t))\sin(\alpha(t))\bigl(-\cos(\alpha(t))\,(C_{\mathrm{L0}} + C_{\mathrm{L}\alpha}\,\alpha(t)) +$$

$$\cos(\beta(t))\sin(\alpha(t))\bigl(-K_{\mathrm{CD}}\,(C_{\mathrm{L0}} + C_{\mathrm{L}\alpha}\,\alpha(t))^2 - C_{\mathrm{D0}}\bigr) - \sin(\alpha(t))\sin(\beta(t))\,C_{\mathrm{Y}\beta}\,\beta(t)\bigr)(t)\,\beta'(t) -$$

$$2.\cos(\alpha(t))\sin(\beta(t))\bigl(\sin(\alpha(t))\,C_{\mathrm{L}\alpha}\,\alpha'(t) + \cos(\alpha(t))\,(C_{\mathrm{L0}} + C_{\mathrm{L}\alpha}\,\alpha(t))\,\alpha'(t) - 2\cos(\alpha(t))\cos(\beta(t))$$

$$C_{\mathrm{L}\alpha}\,K_{\mathrm{CD}}\,(C_{\mathrm{L0}} + C_{\mathrm{L}\alpha}\,\alpha(t))\,\alpha'(t) - \cos(\beta(t))\sin(\alpha(t))\bigl(-K_{\mathrm{CD}}\,(C_{\mathrm{L0}} + C_{\mathrm{L}\alpha}\,\alpha(t))^2 - C_{\mathrm{D0}}\bigr)\,\alpha'(t) +$$

$$\sin(\alpha(t))\sin(\beta(t))\,C_{\mathrm{Y}\beta}\,\beta(t)\,\alpha'(t) - \cos(\alpha(t))\sin(\beta(t))\,C_{\mathrm{Y}\beta}\,\beta'(t) -$$

$$\cos(\alpha(t))\sin(\beta(t))\bigl(-K_{\mathrm{CD}}\,(C_{\mathrm{L0}} + C_{\mathrm{L}\alpha}\,\alpha(t))^2 - C_{\mathrm{D0}}\bigr)\,\beta'(t) - \cos(\alpha(t))\cos(\beta(t))\,C_{\mathrm{Y}\beta}\,\beta(t)\,\beta'(t)\bigr)(t) -$$

$$2.\sin(\alpha(t))\sin(\beta(t))\bigl(-\cos(\alpha(t))\,C_{\mathrm{L}\alpha}\,\alpha'(t) + \sin(\alpha(t))\,(C_{\mathrm{L0}} + C_{\mathrm{L}\alpha}\,\alpha(t))\,\alpha'(t) - 2\cos(\beta(t))\sin(\alpha(t))$$

$$C_{\mathrm{L}\alpha}\,K_{\mathrm{CD}}\,(C_{\mathrm{L0}} + C_{\mathrm{L}\alpha}\,\alpha(t))\,\alpha'(t) + \cos(\alpha(t))\cos(\beta(t))\bigl(-K_{\mathrm{CD}}\,(C_{\mathrm{L0}} + C_{\mathrm{L}\alpha}\,\alpha(t))^2 - C_{\mathrm{D0}}\bigr)\,\alpha'(t) -$$

$$\cos(\alpha(t))\sin(\beta(t))\,C_{\mathrm{Y}\beta}\,\beta(t)\,\alpha'(t) - \sin(\alpha(t))\sin(\beta(t))\,C_{\mathrm{Y}\beta}\,\beta'(t) -$$

$$\sin(\alpha(t))\sin(\beta(t))\bigl(-K_{\mathrm{CD}}\,(C_{\mathrm{L0}} + C_{\mathrm{L}\alpha}\,\alpha(t))^2 - C_{\mathrm{D0}}\bigr)\,\beta'(t) - \cos(\beta(t))\sin(\alpha(t))\,C_{\mathrm{Y}\beta}\,\beta(t)\,\beta'(t)\bigr)(t) +$$

$$2\cos(\beta(t))\bigl(-2\sin(\beta(t))\,C_{\mathrm{L}\alpha}\,K_{\mathrm{CD}}\,(C_{\mathrm{L0}} + C_{\mathrm{L}\alpha}\,\alpha(t))\,\alpha'(t) + \cos(\beta(t))\,C_{\mathrm{Y}\beta}\,\beta'(t) +$$

$$\cos(\beta(t))\bigl(-K_{\mathrm{CD}}\,(C_{\mathrm{L0}} + C_{\mathrm{L}\alpha}\,\alpha(t))^2 - C_{\mathrm{D0}}\bigr)\,\beta'(t) - \sin(\beta(t))\,C_{\mathrm{Y}\beta}\,\beta(t)\,\beta'(t)\bigr)(t)\bigr)\,V(t)^2 +$$

$$1.\,S\,\rho\,\bigl(\cos(\beta(t))\bigl(\sin(\beta(t))\bigl(-K_{\mathrm{CD}}\,(C_{\mathrm{L0}} + C_{\mathrm{L}\alpha}\,\alpha(t))^2 - C_{\mathrm{D0}}\bigr) + \cos(\beta(t))\,C_{\mathrm{Y}\beta}\,\beta(t)\bigr)(t) -$$

$$1.\cos(\alpha(t))\sin(\beta(t))\bigl(\sin(\alpha(t))\,(C_{\mathrm{L0}} + C_{\mathrm{L}\alpha}\,\alpha(t)) + \cos(\alpha(t))\cos(\beta(t))\bigl(-K_{\mathrm{CD}}\,(C_{\mathrm{L0}} + C_{\mathrm{L}\alpha}\,\alpha(t))^2 - C_{\mathrm{D0}}\bigr) -$$

$$\cos(\alpha(t))\sin(\beta(t))\,C_{\mathrm{Y}\beta}\,\beta(t)\bigr)(t) - 1.\sin(\alpha(t))\sin(\beta(t))\bigl(-\cos(\alpha(t))\,(C_{\mathrm{L0}} + C_{\mathrm{L}\alpha}\,\alpha(t)) +$$

$$\cos(\beta(t))\sin(\alpha(t))\bigl(-K_{\mathrm{CD}}\,(C_{\mathrm{L0}} + C_{\mathrm{L}\alpha}\,\alpha(t))^2 - C_{\mathrm{D0}}\bigr) - \sin(\alpha(t))\sin(\beta(t))\,C_{\mathrm{Y}\beta}\,\beta(t)\bigr)(t)\bigr)\,V'(t)\,V(t) +$$

$$1.\,m\,(\sin(\alpha(t))\,p'(t) - 1.\cos(\alpha(t))\,r'(t) + \cos(\alpha(t))\,p(t)\,\alpha'(t) + 1.\,r(t)\sin(\alpha(t))\,\alpha'(t))$$

$$V(t) + 1.$$

$$\cos(\alpha(t))$$

$$\sin(\beta(t))\,T'(t) +$$

$$1.\,m\,(p(t)\sin(\alpha(t)) - 1.\cos(\alpha(t))\,r(t))\,V'(t) -$$

$$1.\sin(\alpha(t))\sin(\beta(t))\,T(t)\,\alpha'(t) +$$

$$1.\cos(\alpha(t))\cos(\beta(t))\,T(t)\,\beta'(t) +$$

$$1.\,g\,m\,(-1.\cos(\alpha(t))\cos(\theta(t))\cos(\phi(t))\sin(\beta(t))\,\alpha'(t) - \sin(\alpha(t))\sin(\beta(t))\sin(\theta(t))\,\alpha'(t) -$$

$$1.\cos(\beta(t))\cos(\theta(t))\cos(\phi(t))\sin(\alpha(t))\,\beta'(t) + \cos(\alpha(t))\cos(\beta(t))\sin(\theta(t))\,\beta'(t) -$$

$$\cos(\theta(t))\sin(\beta(t))\sin(\phi(t))\,\beta'(t) + \cos(\alpha(t))\cos(\theta(t))\sin(\beta(t))\,\theta'(t) +$$

$$1.\cos(\phi(t))\sin(\alpha(t))\sin(\beta(t))\sin(\theta(t))\,\theta'(t) - \cos(\beta(t))\sin(\theta(t))\sin(\phi(t))\,\theta'(t) +$$

$$\cos(\beta(t))\cos(\theta(t))\cos(\phi(t))\,\phi'(t) + 1.\cos(\theta(t))\sin(\alpha(t))\sin(\beta(t))\sin(\phi(t))\,\phi'(t))\bigr) -$$

$$\frac{1}{m\,V(t)^2}\bigl(0.5\,S\,\rho\,\bigl(\cos(\beta(t))\bigl(\sin(\beta(t))\bigl(-K_{\mathrm{CD}}\,(C_{\mathrm{L0}} + C_{\mathrm{L}\alpha}\,\alpha(t))^2 - C_{\mathrm{D0}}\bigr) + \cos(\beta(t))\,C_{\mathrm{Y}\beta}\,\beta(t)\bigr)(t) -$$

$$1.\cos(\alpha(t))\sin(\beta(t))\bigl(\sin(\alpha(t))\,(C_{\mathrm{L0}} + C_{\mathrm{L}\alpha}\,\alpha(t)) + \cos(\alpha(t))\cos(\beta(t))\bigl(-K_{\mathrm{CD}}\,(C_{\mathrm{L0}} + C_{\mathrm{L}\alpha}\,\alpha(t))^2 - C_{\mathrm{D0}}\bigr) -$$

$$\cos(\alpha(t))\sin(\beta(t))\,C_{\mathrm{Y}\beta}\,\beta(t)\bigr)(t) - 1.\sin(\alpha(t))\sin(\beta(t))\bigl(-\cos(\alpha(t))\,(C_{\mathrm{L0}} + C_{\mathrm{L}\alpha}\,\alpha(t)) +$$

$$\cos(\beta(t))\sin(\alpha(t))\bigl(-K_{\mathrm{CD}}\,(C_{\mathrm{L0}} + C_{\mathrm{L}\alpha}\,\alpha(t))^2 - C_{\mathrm{D0}}\bigr) - \sin(\alpha(t))\sin(\beta(t))\,C_{\mathrm{Y}\beta}\,\beta(t)\bigr)(t)\bigr)\,V(t)^2 +$$

$$1.\,m\,(p(t)\sin(\alpha(t)) - 1.\cos(\alpha(t))\,r(t))\,V(t) + 1.\,g\,m\,(-1.\cos(\theta(t))\cos(\phi(t))\sin(\alpha(t))\sin(\beta(t)) +$$

$$\cos(\alpha(t))\sin(\theta(t))\sin(\beta(t)) + \cos(\beta(t))\cos(\theta(t))\sin(\phi(t))) + 1.\cos(\alpha(t))\sin(\beta(t))\,T(t)\bigr)\,V'(t)\}\}$$

$$\Big\{\Big\{\alpha''(t) \to$$

$$-\frac{1}{m\, V(t)^2} \sec(\beta(t)) \big(0.5\, S\, \rho \big(\cos(\alpha(t)) \big(-\cos(\alpha(t))\, (C_{\mathrm{L0}} + C_{\mathrm{L}\alpha}\, \alpha(t)) + \cos(\beta(t)) \sin(\alpha(t)) \big(-K_{\mathrm{CD}}\, (C_{\mathrm{L0}} + C_{\mathrm{L}\alpha}\, \alpha(t))^2 - C_{\mathrm{D0}}\big) -$$

$$\sin(\alpha(t)) \sin(\beta(t))\, C_{\mathrm{Y}\beta}\, \beta(t)\big)(t) - 1.\, \sin(\alpha(t)) \big(\sin(\alpha(t))\, (C_{\mathrm{L0}} + C_{\mathrm{L}\alpha}\, \alpha(t)) + \cos(\alpha(t))$$

$$\cos(\beta(t)) \big(-K_{\mathrm{CD}}\, (C_{\mathrm{L0}} + C_{\mathrm{L}\alpha}\, \alpha(t))^2 - C_{\mathrm{D0}}\big) - \cos(\alpha(t)) \sin(\beta(t))\, C_{\mathrm{Y}\beta}\, \beta(t)\big)(t)\big)\, V(t)^2 +$$

$$1.\, m\, (\cos(\beta(t))\, q(t) - 1.\, \cos(\alpha(t))\, p(t) \sin(\beta(t)) - 1.\, r(t) \sin(\alpha(t)) \sin(\beta(t)))\, V(t) +$$

$$1.\, g\, m\, (\cos(\alpha(t)) \cos(\theta(t)) \cos(\phi(t)) + \sin(\alpha(t)) \sin(\theta(t))) + 1.\, \sin(\alpha(t))\, T(t)\big)\, V'(t) + \frac{1}{m\, V(t)} \sec(\beta(t)) \tan(\beta(t))$$

$$\big(0.5\, S\, \rho \big(\cos(\alpha(t)) \big(-\cos(\alpha(t))\, (C_{\mathrm{L0}} + C_{\mathrm{L}\alpha}\, \alpha(t)) + \cos(\beta(t)) \sin(\alpha(t)) \big(-K_{\mathrm{CD}}\, (C_{\mathrm{L0}} + C_{\mathrm{L}\alpha}\, \alpha(t))^2 - C_{\mathrm{D0}}\big) -$$

$$\sin(\alpha(t)) \sin(\beta(t))\, C_{\mathrm{Y}\beta}\, \beta(t)\big)(t) - 1.\, \sin(\alpha(t)) \big(\sin(\alpha(t))\, (C_{\mathrm{L0}} + C_{\mathrm{L}\alpha}\, \alpha(t)) +$$

$$\cos(\alpha(t)) \cos(\beta(t)) \big(-K_{\mathrm{CD}}\, (C_{\mathrm{L0}} + C_{\mathrm{L}\alpha}\, \alpha(t))^2 - C_{\mathrm{D0}}\big) - \cos(\alpha(t)) \sin(\beta(t))\, C_{\mathrm{Y}\beta}\, \beta(t)\big)(t)\big)\, V(t)^2 +$$

$$1.\, m\, (\cos(\beta(t))\, q(t) - 1.\, \cos(\alpha(t))\, p(t) \sin(\beta(t)) - 1.\, r(t) \sin(\alpha(t)) \sin(\beta(t)))\, V(t) +$$

$$1.\, g\, m\, (\cos(\alpha(t)) \cos(\theta(t)) \cos(\phi(t)) + \sin(\alpha(t)) \sin(\theta(t))) + 1.\, \sin(\alpha(t))\, T(t)\big)\, \beta'(t) + \frac{1}{m\, V(t)}$$

$$\sec(\beta(t)) \big(0.5\, S\, \rho \big(-1.\, \cos(\alpha(t)) \big(\sin(\alpha(t))\, (C_{\mathrm{L0}} + C_{\mathrm{L}\alpha}\, \alpha(t)) + \cos(\alpha(t)) \cos(\beta(t)) \big(-K_{\mathrm{CD}}\, (C_{\mathrm{L0}} + C_{\mathrm{L}\alpha}\, \alpha(t))^2 - C_{\mathrm{D0}}\big) -$$

$$\cos(\alpha(t)) \sin(\beta(t))\, C_{\mathrm{Y}\beta}\, \beta(t)\big)(t)\, \alpha'(t) - \sin(\alpha(t)) \big(-\cos(\alpha(t))\, (C_{\mathrm{L0}} + C_{\mathrm{L}\alpha}\, \alpha(t)) +$$

$$\cos(\beta(t)) \sin(\alpha(t)) \big(-K_{\mathrm{CD}}\, (C_{\mathrm{L0}} + C_{\mathrm{L}\alpha}\, \alpha(t))^2 - C_{\mathrm{D0}}\big) - \sin(\alpha(t)) \sin(\beta(t))\, C_{\mathrm{Y}\beta}\, \beta(t)\big)(t)\, \alpha'(t) -$$

$$2.\, \sin(\alpha(t)) \big(\sin(\alpha(t))\, C_{\mathrm{L}\alpha}\, \alpha'(t) + \cos(\alpha(t))\, (C_{\mathrm{L0}} + C_{\mathrm{L}\alpha}\, \alpha(t))\, \alpha'(t) - 2 \cos(\alpha(t)) \cos(\beta(t))\, C_{\mathrm{L}\alpha}$$

$$K_{\mathrm{CD}}\, (C_{\mathrm{L0}} + C_{\mathrm{L}\alpha}\, \alpha(t))\, \alpha'(t) - \cos(\beta(t)) \sin(\alpha(t)) \big(-K_{\mathrm{CD}}\, (C_{\mathrm{L0}} + C_{\mathrm{L}\alpha}\, \alpha(t))^2 - C_{\mathrm{D0}}\big)\, \alpha'(t) +$$

$$\sin(\alpha(t)) \sin(\beta(t))\, C_{\mathrm{Y}\beta}\, \beta(t)\, \alpha'(t) - \cos(\alpha(t)) \sin(\beta(t))\, C_{\mathrm{Y}\beta}\, \beta'(t) -$$

$$\cos(\alpha(t)) \sin(\beta(t)) \big(-K_{\mathrm{CD}}\, (C_{\mathrm{L0}} + C_{\mathrm{L}\alpha}\, \alpha(t))^2 - C_{\mathrm{D0}}\big)\, \beta'(t) - \cos(\alpha(t)) \cos(\beta(t))\, C_{\mathrm{Y}\beta}\, \beta(t)\, \beta'(t)\big)(t) +$$

$$2 \cos(\alpha(t)) \big(-\cos(\alpha(t))\, C_{\mathrm{L}\alpha}\, \alpha'(t) + \sin(\alpha(t))\, (C_{\mathrm{L0}} + C_{\mathrm{L}\alpha}\, \alpha(t))\, \alpha'(t) - 2 \cos(\beta(t)) \sin(\alpha(t))\, C_{\mathrm{L}\alpha}$$

$$K_{\mathrm{CD}}\, (C_{\mathrm{L0}} + C_{\mathrm{L}\alpha}\, \alpha(t))\, \alpha'(t) + \cos(\alpha(t)) \cos(\beta(t)) \big(-K_{\mathrm{CD}}\, (C_{\mathrm{L0}} + C_{\mathrm{L}\alpha}\, \alpha(t))^2 - C_{\mathrm{D0}}\big)\, \alpha'(t) -$$

$$\cos(\alpha(t)) \sin(\beta(t))\, C_{\mathrm{Y}\beta}\, \beta(t)\, \alpha'(t) - \sin(\alpha(t)) \sin(\beta(t))\, C_{\mathrm{Y}\beta}\, \beta'(t) - \sin(\alpha(t)) \sin(\beta(t))$$

$$\big(-K_{\mathrm{CD}}\, (C_{\mathrm{L0}} + C_{\mathrm{L}\alpha}\, \alpha(t))^2 - C_{\mathrm{D0}}\big)\, \beta'(t) - \cos(\beta(t)) \sin(\alpha(t))\, C_{\mathrm{Y}\beta}\, \beta(t)\, \beta'(t)\big)(t)\big)\, V(t)^2 +$$

$$1.\, S\, \rho \big(\cos(\alpha(t)) \big(-\cos(\alpha(t))\, (C_{\mathrm{L0}} + C_{\mathrm{L}\alpha}\, \alpha(t)) + \cos(\beta(t)) \sin(\alpha(t)) \big(-K_{\mathrm{CD}}\, (C_{\mathrm{L0}} + C_{\mathrm{L}\alpha}\, \alpha(t))^2 - C_{\mathrm{D0}}\big) -$$

$$\sin(\alpha(t)) \sin(\beta(t))\, C_{\mathrm{Y}\beta}\, \beta(t)\big)(t) - 1.\, \sin(\alpha(t)) \big(\sin(\alpha(t))\, (C_{\mathrm{L0}} + C_{\mathrm{L}\alpha}\, \alpha(t)) +$$

$$\cos(\alpha(t)) \cos(\beta(t)) \big(-K_{\mathrm{CD}}\, (C_{\mathrm{L0}} + C_{\mathrm{L}\alpha}\, \alpha(t))^2 - C_{\mathrm{D0}}\big) - \cos(\alpha(t)) \sin(\beta(t))\, C_{\mathrm{Y}\beta}\, \beta(t)\big)(t)\big)\, V'(t)\, V(t) +$$

$$1.\, m\, (-1.\, \cos(\alpha(t)) \sin(\beta(t))\, p'(t) + \cos(\beta(t))\, q'(t) - 1.\, \sin(\alpha(t)) \sin(\beta(t))\, r'(t) -$$

$$1.\, \cos(\alpha(t))\, r(t) \sin(\beta(t))\, \alpha'(t) + 1.\, p(t) \sin(\alpha(t)) \sin(\beta(t))\, \alpha'(t) -$$

$$1.\, \cos(\alpha(t)) \cos(\beta(t))\, p(t)\, \beta'(t) - 1.\, \cos(\beta(t))\, r(t) \sin(\alpha(t))\, \beta'(t) - q(t) \sin(\beta(t))\, \beta'(t))\, V(t) +$$

$$1.\, \sin(\alpha(t))\, T'(t) + 1.\, m\, (\cos(\beta(t))\, q(t) - 1.\, \cos(\alpha(t))\, p(t) \sin(\beta(t)) - 1.\, r(t) \sin(\alpha(t)) \sin(\beta(t)))\, V'(t) +$$

$$1.\, \cos(\alpha(t))\, T(t)\, \alpha'(t) +$$

$$1.\, g\, m\, (-\cos(\theta(t)) \cos(\phi(t)) \sin(\alpha(t))\, \alpha'(t) + \cos(\alpha(t)) \sin(\theta(t))\, \alpha'(t) + \cos(\theta(t)) \sin(\alpha(t))\, \theta'(t) -$$

$$\cos(\alpha(t)) \cos(\phi(t)) \sin(\theta(t))\, \theta'(t) - \cos(\alpha(t)) \cos(\theta(t)) \sin(\phi(t))\, \phi'(t))\big)\Big\}\Big\}$$

$$
\begin{aligned}
&\{\{\psi''(t) \to \tan(\theta_w(t))\,\theta_w{}'(t)\,(\psi'(t) - \psi_w{}'(t))\sec^2(\psi(t) - \psi_w(t)) - \cos(\beta(t))\sec(\theta_w(t))\sin(\alpha(t))\sin(\phi(t))\,\alpha'(t)^2\sec(\psi(t) - \psi_w(t)) + \\
&\quad \cos(\phi(t))\sec(\theta_w(t))\sin(\beta(t))\,\beta'(t)^2\sec(\psi(t) - \psi_w(t)) - \cos(\beta(t))\sec(\theta_w(t))\sin(\alpha(t))\sin(\phi(t))\,\beta'(t)^2\sec(\psi(t) - \psi_w(t)) + \\
&\quad \cos(\phi(t))\sec(\theta_w(t))\sin(\beta(t))\,\phi'(t)^2\sec(\psi(t) - \psi_w(t)) - \cos(\beta(t))\sec(\theta_w(t))\sin(\alpha(t))\sin(\phi(t))\,\phi'(t)^2\sec(\psi(t) - \psi_w(t)) - \\
&\quad 2\cos(\alpha(t))\sec(\theta_w(t))\sin(\beta(t))\sin(\phi(t))\,\alpha'(t)\,\beta'(t)\sec(\psi(t) - \psi_w(t)) + 2\cos(\alpha(t))\cos(\beta(t))\cos(\phi(t))\sec(\theta_w(t)) \\
&\qquad \alpha'(t)\,\phi'(t)\sec(\psi(t) - \psi_w(t)) - 2\cos(\phi(t))\sec(\theta_w(t))\sin(\alpha(t))\sin(\beta(t))\,\beta'(t)\,\phi'(t)\sec(\psi(t) - \psi_w(t)) + \\
&\quad 2\cos(\beta(t))\sec(\theta_w(t))\sin(\phi(t))\,\beta'(t)\,\phi'(t)\sec(\psi(t) - \psi_w(t)) + \cos(\alpha(t))\cos(\beta(t))\sec(\theta_w(t))\sin(\phi(t))\tan(\theta_w(t)) \\
&\qquad \alpha'(t)\,\theta_w{}'(t)\sec(\psi(t) - \psi_w(t)) - \cos(\beta(t))\cos(\phi(t))\sec(\theta_w(t))\tan(\theta_w(t))\,\beta'(t)\,\theta_w{}'(t)\sec(\psi(t) - \psi_w(t)) - \\
&\quad \sec(\theta_w(t))\sin(\alpha(t))\sin(\beta(t))\sin(\phi(t))\tan(\theta_w(t))\,\beta'(t)\,\theta_w{}'(t)\sec(\psi(t) - \psi_w(t)) + \\
&\quad \cos(\beta(t))\cos(\phi(t))\sec(\theta_w(t))\sin(\alpha(t))\tan(\theta_w(t))\,\phi'(t)\,\theta_w{}'(t)\sec(\psi(t) - \psi_w(t)) + \\
&\quad \sec(\theta_w(t))\sin(\beta(t))\sin(\phi(t))\tan(\theta_w(t))\,\phi'(t)\,\theta_w{}'(t)\sec(\psi(t) - \psi_w(t)) + \\
&\quad \cos(\alpha(t))\cos(\beta(t))\sec(\theta_w(t))\sin(\phi(t))\tan(\psi(t) - \psi_w(t))\,\alpha'(t)\,(\psi'(t) - \psi_w{}'(t))\sec(\psi(t) - \psi_w(t)) - \\
&\quad \cos(\beta(t))\cos(\phi(t))\sec(\theta_w(t))\tan(\psi(t) - \psi_w(t))\,\beta'(t)\,(\psi'(t) - \psi_w{}'(t))\sec(\psi(t) - \psi_w(t)) - \\
&\quad \sec(\theta_w(t))\sin(\alpha(t))\sin(\beta(t))\sin(\phi(t))\tan(\psi(t) - \psi_w(t))\,\beta'(t)\,(\psi'(t) - \psi_w{}'(t))\sec(\psi(t) - \psi_w(t)) + \\
&\quad \cos(\beta(t))\cos(\phi(t))\sec(\theta_w(t))\sin(\alpha(t))\tan(\psi(t) - \psi_w(t))\,\phi'(t)\,(\psi'(t) - \psi_w{}'(t))\sec(\psi(t) - \psi_w(t)) + \\
&\quad \sec(\theta_w(t))\sin(\beta(t))\sin(\phi(t))\tan(\psi(t) - \psi_w(t))\,\phi'(t)\,(\psi'(t) - \psi_w{}'(t))\sec(\psi(t) - \psi_w(t)) + \\
&\quad \cos(\alpha(t))\cos(\beta(t))\sec(\theta_w(t))\sin(\phi(t))\,\alpha''(t)\sec(\psi(t) - \psi_w(t)) - \\
&\quad \cos(\beta(t))\cos(\phi(t))\sec(\theta_w(t))\,\beta''(t)\sec(\psi(t) - \psi_w(t)) - \sec(\theta_w(t))\sin(\alpha(t))\sin(\beta(t))\sin(\phi(t))\,\beta''(t)\sec(\psi(t) - \psi_w(t)) + \\
&\quad \cos(\beta(t))\cos(\phi(t))\sec(\theta_w(t))\sin(\alpha(t))\,\phi''(t)\sec(\psi(t) - \psi_w(t)) + \sec(\theta_w(t))\sin(\beta(t))\sin(\phi(t))\,\phi''(t)\sec(\psi(t) - \psi_w(t)) + \\
&\quad \sec^2(\theta_w(t))\tan(\psi(t) - \psi_w(t))\,\theta_w{}'(t)^2 + \tan(\theta_w(t))\tan(\psi(t) - \psi_w(t))\,\theta_w{}''(t) + \psi_w{}''(t)\}\}
\end{aligned}
$$

$$
\begin{aligned}
\{\{\Theta'' \to \; & -\Big(\big((-\mathrm{cB}\,\mathrm{cT}\,\mathrm{sA}\,\alpha' - \mathrm{cA}\,\mathrm{cB}\,\mathrm{cF}\,\mathrm{sT}\,\alpha' - \mathrm{cA}\,\mathrm{cT}\,\mathrm{sB}\,\beta' + \mathrm{cF}\,\mathrm{sA}\,\mathrm{sB}\,\mathrm{sT}\,\beta' + \mathrm{cB}\,\mathrm{sF}\,\mathrm{sT}\,\beta' - \mathrm{cB}\,\mathrm{cF}\,\mathrm{cT}\,\mathrm{sA}\,\Theta' + \mathrm{cT}\,\mathrm{sB} \\
& \quad \mathrm{sF}\,\Theta' - \mathrm{cA}\,\mathrm{cB}\,\mathrm{sT}\,\Theta' + \mathrm{cF}\,\mathrm{sB}\,\mathrm{sT}\,\phi' + \mathrm{cB}\,\mathrm{sA}\,\mathrm{sF}\,\mathrm{sT}\,\phi') \\
& \quad (-\mathrm{cA}\,\mathrm{cB}\,\mathrm{cF}\,\mathrm{cT}\,\alpha' + \mathrm{cB}\,\mathrm{sA}\,\mathrm{sT}\,\alpha' + \mathrm{cF}\,\mathrm{cT}\,\mathrm{sA}\,\mathrm{sB}\,\beta' + \mathrm{cB}\,\mathrm{cT}\,\mathrm{sF}\,\beta' + \mathrm{cA}\,\mathrm{sB}\,\mathrm{sT}\,\beta' + \\
& \quad \mathrm{cF}\,\mathrm{cT}\,\mathrm{sB}\,\phi' + \mathrm{cB}\,\mathrm{cT}\,\mathrm{sA}\,\mathrm{sF}\,\phi' + \mathrm{cPw}\,\psi_{\mathrm{w}}') \big) \Big/ (\mathrm{cA}\,\mathrm{cB}\,\mathrm{cT} - \mathrm{cB}\,\mathrm{cF}\,\mathrm{sA}\,\mathrm{sT} + \mathrm{sB}\,\mathrm{sF}\,\mathrm{sT})^2 \Big) + \\
& \Big(\mathrm{cB}\,\mathrm{cF}\,\mathrm{cT}\,\mathrm{sA}\,(\alpha')^2 + \mathrm{cA}\,\mathrm{cB}\,\mathrm{sT}\,(\alpha')^2 + 2\,\mathrm{cA}\,\mathrm{cF}\,\mathrm{cT}\,\mathrm{sB}\,\alpha'\,\beta' - 2\,\mathrm{sA}\,\mathrm{sB}\,\mathrm{sT}\,\alpha'\,\beta' + \mathrm{cB}\,\mathrm{cF}\,\mathrm{cT}\,\mathrm{sA}\,(\beta')^2 - \\
& \quad \mathrm{cT}\,\mathrm{sB}\,\mathrm{sF}\,(\beta')^2 + \mathrm{cA}\,\mathrm{cB}\,\mathrm{sT}\,(\beta')^2 + \mathrm{cB}\,\mathrm{cT}\,\mathrm{sA}\,\alpha'\,\Theta' + \mathrm{cA}\,\mathrm{cB}\,\mathrm{cF}\,\mathrm{sT}\,\alpha'\,\Theta' + \mathrm{cA}\,\mathrm{cT}\,\mathrm{sB}\,\beta'\,\Theta' - \\
& \quad \mathrm{cF}\,\mathrm{sA}\,\mathrm{sB}\,\mathrm{sT}\,\beta'\,\Theta' - \mathrm{cB}\,\mathrm{sF}\,\mathrm{sT}\,\beta'\,\Theta' + 2\,\mathrm{cA}\,\mathrm{cB}\,\mathrm{cT}\,\mathrm{sF}\,\alpha'\,\phi' + 2\,\mathrm{cB}\,\mathrm{cF}\,\mathrm{cT}\,\beta'\,\phi' - 2\,\mathrm{cT}\,\mathrm{sA}\,\mathrm{sB}\,\mathrm{sF}\,\beta'\,\phi' - \\
& \quad \mathrm{cF}\,\mathrm{sB}\,\mathrm{sT}\,\Theta'\,\phi' - \mathrm{cB}\,\mathrm{sA}\,\mathrm{sF}\,\mathrm{sT}\,\Theta'\,\phi' + \mathrm{cB}\,\mathrm{cF}\,\mathrm{cT}\,\mathrm{sA}\,(\phi')^2 - \mathrm{cT}\,\mathrm{sB}\,\mathrm{sF}\,(\phi')^2 - \mathrm{sPw}\,(\psi_{\mathrm{w}}')^2 - \\
& \quad \mathrm{cA}\,\mathrm{cB}\,\mathrm{cF}\,\mathrm{cT}\,\alpha'' + \mathrm{cB}\,\mathrm{sA}\,\mathrm{sT}\,\alpha'' + \mathrm{cF}\,\mathrm{cT}\,\mathrm{sA}\,\mathrm{sB}\,\beta'' + \mathrm{cB}\,\mathrm{cT}\,\mathrm{sF}\,\beta'' + \mathrm{cA}\,\mathrm{sB}\,\mathrm{sT}\,\beta'' + \\
& \quad \mathrm{cF}\,\mathrm{cT}\,\mathrm{sB}\,\phi'' + \mathrm{cB}\,\mathrm{cT}\,\mathrm{sA}\,\mathrm{sF}\,\phi'' + \mathrm{cPw}\,\psi_{\mathrm{w}}'' \Big) \Big/ (\mathrm{cA}\,\mathrm{cB}\,\mathrm{cT} - \mathrm{cB}\,\mathrm{cF}\,\mathrm{sA}\,\mathrm{sT} + \mathrm{sB}\,\mathrm{sF}\,\mathrm{sT}) \}\}
\end{aligned}
$$

Out[642]//TraditionalForm=

$$
\begin{aligned}
\{\{\theta''(t) \to \; & \big(\cos(\beta(t))\cos(\theta(t))\cos(\phi(t))\sin(\alpha(t))\,\alpha'(t)^2 + \cos(\alpha(t))\cos(\beta(t))\sin(\theta(t))\,\alpha'(t)^2 + 2\cos(\alpha(t))\cos(\theta(t))\cos(\phi(t))\sin(\beta(t)) \\
& \quad \beta'(t)\,\alpha'(t) - 2\sin(\alpha(t))\sin(\beta(t))\sin(\theta(t))\,\beta'(t)\,\alpha'(t) + \cos(\beta(t))\cos(\theta(t))\sin(\alpha(t))\,\theta'(t)\,\alpha'(t) + \\
& \quad \cos(\alpha(t))\cos(\beta(t))\cos(\phi(t))\sin(\theta(t))\,\theta'(t)\,\alpha'(t) + 2\cos(\alpha(t))\cos(\beta(t))\cos(\theta(t))\sin(\phi(t))\,\phi'(t)\,\alpha'(t) + \\
& \quad \cos(\beta(t))\cos(\theta(t))\cos(\phi(t))\sin(\alpha(t))\,\beta'(t)^2 + \cos(\alpha(t))\cos(\beta(t))\sin(\theta(t))\,\beta'(t)^2 - \\
& \quad \cos(\theta(t))\sin(\beta(t))\sin(\phi(t))\,\beta'(t)^2 + \cos(\beta(t))\cos(\theta(t))\cos(\phi(t))\sin(\alpha(t))\,\phi'(t)^2 - \\
& \quad \cos(\theta(t))\sin(\beta(t))\sin(\phi(t))\,\phi'(t)^2 - \sin(\psi_w(t))\,\psi_w{}'(t)^2 + \cos(\alpha(t))\cos(\theta(t))\sin(\beta(t))\,\beta'(t)\,\theta'(t) - \\
& \quad \cos(\phi(t))\sin(\alpha(t))\sin(\beta(t))\sin(\theta(t))\,\beta'(t)\,\theta'(t) - \cos(\beta(t))\sin(\theta(t))\sin(\phi(t))\,\beta'(t)\,\theta'(t) + \\
& \quad 2\cos(\beta(t))\cos(\theta(t))\cos(\phi(t))\,\beta'(t)\,\phi'(t) - 2\cos(\theta(t))\sin(\alpha(t))\sin(\beta(t))\sin(\phi(t))\,\beta'(t)\,\phi'(t) - \\
& \quad \cos(\phi(t))\sin(\beta(t))\sin(\theta(t))\,\theta'(t)\,\phi'(t) - \cos(\beta(t))\sin(\alpha(t))\sin(\theta(t))\sin(\phi(t))\,\theta'(t)\,\phi'(t) - \\
& \quad \cos(\alpha(t))\cos(\beta(t))\cos(\theta(t))\cos(\phi(t))\,\alpha''(t) + \cos(\beta(t))\sin(\alpha(t))\sin(\theta(t))\,\alpha''(t) + \\
& \quad \cos(\theta(t))\cos(\phi(t))\sin(\alpha(t))\sin(\beta(t))\,\beta''(t) + \cos(\alpha(t))\sin(\beta(t))\sin(\theta(t))\,\beta''(t) + \cos(\beta(t))\cos(\theta(t))\sin(\phi(t))\,\beta''(t) + \\
& \quad \cos(\theta(t))\cos(\phi(t))\sin(\beta(t))\,\phi''(t) + \cos(\beta(t))\cos(\theta(t))\sin(\alpha(t))\sin(\phi(t))\,\phi''(t) + \cos(\psi_w(t))\,\psi_w{}''(t)\big) \Big/ \\
& (\cos(\alpha(t))\cos(\beta(t))\cos(\theta(t)) - \cos(\beta(t))\cos(\phi(t))\sin(\alpha(t))\sin(\theta(t)) + \sin(\beta(t))\sin(\theta(t))\sin(\phi(t))) - \\
& ((-\cos(\beta(t))\cos(\theta(t))\sin(\alpha(t))\,\alpha'(t) - \cos(\alpha(t))\cos(\beta(t))\cos(\phi(t))\sin(\theta(t))\,\alpha'(t) - \cos(\alpha(t))\cos(\theta(t))\sin(\beta(t))\,\beta'(t) + \\
& \quad \cos(\phi(t))\sin(\alpha(t))\sin(\beta(t))\sin(\theta(t))\,\beta'(t) + \cos(\beta(t))\sin(\theta(t))\sin(\phi(t))\,\beta'(t) - \\
& \quad \cos(\beta(t))\cos(\theta(t))\cos(\phi(t))\sin(\alpha(t))\,\theta'(t) - \cos(\alpha(t))\cos(\beta(t))\sin(\theta(t))\,\theta'(t) + \\
& \quad \cos(\theta(t))\sin(\beta(t))\sin(\phi(t))\,\theta'(t) + \cos(\phi(t))\sin(\beta(t))\sin(\theta(t))\,\phi'(t) + \cos(\beta(t))\sin(\alpha(t))\sin(\theta(t))\sin(\phi(t))\,\phi'(t)) \\
& \quad (-\cos(\alpha(t))\cos(\beta(t))\cos(\theta(t))\cos(\phi(t))\,\alpha'(t) + \cos(\beta(t))\sin(\alpha(t))\sin(\theta(t))\,\alpha'(t) + \cos(\theta(t))\cos(\phi(t)) \\
& \quad \sin(\alpha(t))\sin(\beta(t))\,\beta'(t) + \cos(\alpha(t))\sin(\beta(t))\sin(\theta(t))\,\beta'(t) + \cos(\beta(t))\cos(\theta(t))\sin(\phi(t))\,\beta'(t) + \\
& \quad \cos(\theta(t))\cos(\phi(t))\sin(\beta(t))\,\phi'(t) + \cos(\beta(t))\cos(\theta(t))\sin(\alpha(t))\sin(\phi(t))\,\phi'(t) + \cos(\psi_w(t))\,\psi_w{}'(t))) \Big/ \\
& (\cos(\alpha(t))\cos(\beta(t))\cos(\theta(t)) - \cos(\beta(t))\cos(\phi(t))\sin(\alpha(t))\sin(\theta(t)) + \sin(\beta(t))\sin(\theta(t))\sin(\phi(t)))^2\}\}
\end{aligned}
$$
